# Supplementary material for: Changes in Heart Rate, Heart Rate Variability, Breathing Rate, and Skin Temperature throughout Pregnancy and the Impact of Emotions—A Longitudinal Evaluation Using a Sensor Bracelet
Source: Sensors (Basel). 2023 Jul 23;23(14):6620. doi: 10.3390/s23146620 (PMC10385491; doi:10.3390/s23146620)

Heart rate (beats per minute)

**Model 1: anxiety**	
Gestational age (weeks)	**1.46 [0.77, 1.98]**
Anxious (sometimes to always)	-0.44 [-0.88, 0.02]
**Model 2: stress**	
Gestational age (weeks)	**1.42 [0.79, 1.9]**
Stressed (most of the time to always)	**-3.01 [-4.48, -1.55]**
Gestational age * stressed	**0.13 [0.06, 0.19]**
**Model 3: tiredness**	
Gestational age (weeks)	**1.44 [0.73, 1.96]**
Tired (most of the time to always)	-0.22 [-0.77, 0.5]
**Model 4: sensitivity**	
Gestational age (weeks)	**1.31 [0.63, 1.81]**
Sensitive (most of the time to always)	**-1.2 [-1.99, -0.4]**
**Model 5: unmotivated**	
Gestational age (weeks)	**1.42 [0.75, 1.9]**
Unmotivated (most of the time to always)	-0.12 [-0.89, 0.74]
**Model 6: calm**	
Gestational age (weeks)	**1.44 [0.76, 1.94]**
Calm (sometimes to never)	**-2.54 [-4.2, -0.89]**
Gestational age * calm	**0.1 [0.03, 0.18]**
**Model 7: energized**	
Gestational age (weeks)	**2.21 [1.36, 2.9]**
Energized (sometimes to never)	**-3.65 [-5.92, -0.89]**
Gestational age * energized	**0.25 [0.07, 0.38]**
**Model 8: happiness**	
Gestational age (weeks)	**1.42 [0.76, 1.91]**
Happy (sometimes to never)	-0.23 [-0.76, 0.31]

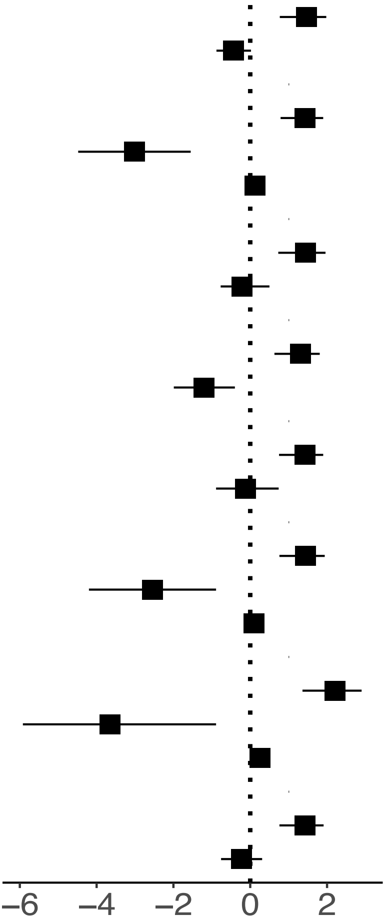

Supplement: Supplementary file 1 [file sensors-23-06620-s001.zip › Suppl. Figure S1a.pdf]
