# Supplementary material for: Changes in Heart Rate, Heart Rate Variability, Breathing Rate, and Skin Temperature throughout Pregnancy and the Impact of Emotions—A Longitudinal Evaluation Using a Sensor Bracelet
Source: Sensors (Basel). 2023 Jul 23;23(14):6620. doi: 10.3390/s23146620 (PMC10385491; doi:10.3390/s23146620)

Heart rate variability

**Model 1: anxiety**	
Gestational age (weeks)	**-3.25 [-4.27, -2.31]**
Anxious (sometimes to always)	-0.34 [-1.2, 0.52]
**Model 2: stress**	
Gestational age (weeks)	**-3.29 [-4.3, -2.39]**
Stressed (most of the time to always)	**6.26 [3.78, 8.64]**
Gestational age * stressed	-0.33 [-0.41, -0.23]
**Model 3: tiredness**	
Gestational age (weeks)	**-3.1 [-4.05, -2.23]**
Tired (most of the time to always)	**-1.09 [-2.05, -0.05]**
**Model 4: sensitivity**	
Gestational age (weeks)	**-3.37 [-4.34, -2.47]**
Sensitive (most of the time to always)	**-4.6 [-8.05, -1.45]**
Gestational age * sensitive	**0.29 [0.12, 0.47]**
**Model 5: unmotivated**	
Gestational age (weeks)	**-3.18 [-4.19, -2.28]**
Unmotivated (most of the time to always)	-1.08 [-2.64, 0.6]
**Model 6: calm**	
Gestational age (weeks)	**-3.29 [-4.32, -2.39]**
Calm (sometimes to never)	2.43 [-0.64, 5.45]
Gestational age * calm	**-0.16 [-0.29, -0.02]**
**Model 7: energized**	
Gestational age (weeks)	**-4.09 [-5.28, -2.84]**
Energized (sometimes to never)	-0.69 [-2.4, 0.94]
**Model 8: happiness**	
Gestational age (weeks)	**-3.04 [-4.11, -2.1]**
Happy (sometimes to never)	**3.61 [1.01, 5.92]**
Gestational age * happy	**-0.2 [-0.3, -0.09]**

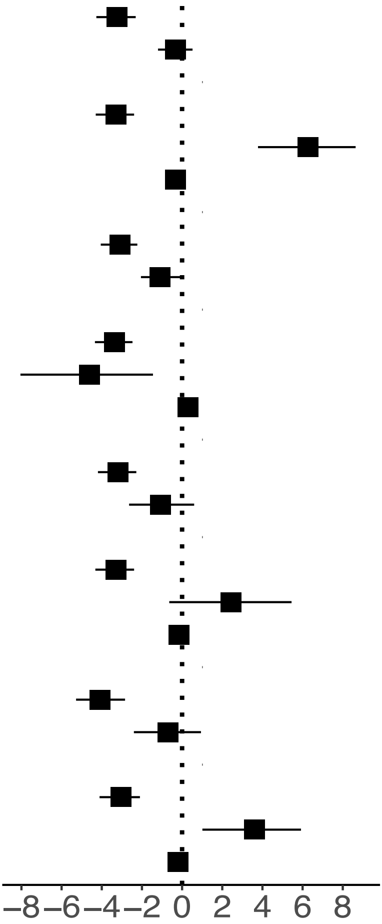

Supplement: Supplementary file 1 [file sensors-23-06620-s001.zip › Suppl. Figure S1b.pdf]
