# Supplementary material for: Changes in Heart Rate, Heart Rate Variability, Breathing Rate, and Skin Temperature throughout Pregnancy and the Impact of Emotions—A Longitudinal Evaluation Using a Sensor Bracelet
Source: Sensors (Basel). 2023 Jul 23;23(14):6620. doi: 10.3390/s23146620 (PMC10385491; doi:10.3390/s23146620)

Breathing rate (breaths per minute)

**Model 1: anxiety**	
Gestational age (weeks)	**-0.15 [-0.23, -0.07]**
Anxious (sometimes to always)	**-0.11 [-0.21, -0.02]**
**Model 2: stress**	
Gestational age (weeks)	**-0.15 [-0.24, -0.07]**
Stressed (most of the time to always)	-0.08 [-0.22, 0.06]
Gestational age * stressed	
**Model 3: tiredness**	
Gestational age (weeks)	**-0.17 [-0.25, -0.08]**
Tired (most of the time to always)	-0.3 [-0.62, 0.07]
Gestational age * tired	**0.01 [0, 0.03]**
**Model 4: sensitivity**	
Gestational age (weeks)	**-0.18 [-0.28, -0.1]**
Sensitive (most of the time to always)	**-0.68 [-1.23, -0.08]**
Gestational age * sensitive	**0.03 [0, 0.06]**
**Model 5: unmotivated**	
Gestational age (weeks)	**-0.14 [-0.17, -0.11]**
Unmotivated (most of the time to always)	-0.13 [-0.31, 0.05]
**Model 6: calm**	
Gestational age (weeks)	**-0.15 [-0.23, -0.07]**
Calm (sometimes to never)	0.09 [-0.23, 0.49]
Gestational age * calm	-0.01 [-0.03, 0.01]
**Model 7: energized**	
Gestational age (weeks)	**-0.19 [-0.27, -0.12]**
Energized (sometimes to never)	-0.43 [-0.83, 0.05]
Gestational age * energized	**0.04 [0.01, 0.06]**
**Model 8: happiness**	
Gestational age (weeks)	**-0.14 [-0.23, -0.06]**
Happy (sometimes to never)	**0.34 [0.1, 0.59]**
Gestational age * happy	**-0.02 [-0.03, 0]**

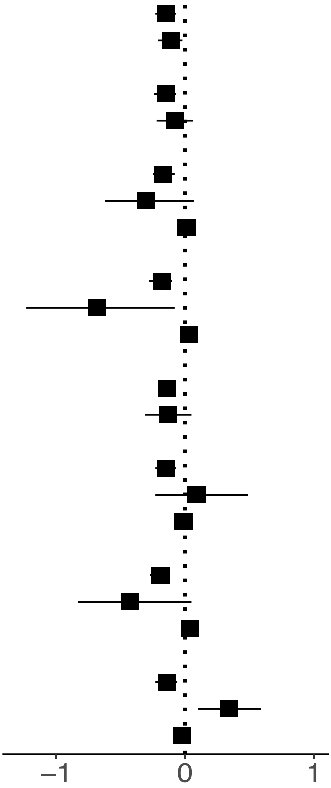

Supplement: Supplementary file 1 [file sensors-23-06620-s001.zip › Suppl. Figure S1c.pdf]
