# Supplementary material for: Changes in Heart Rate, Heart Rate Variability, Breathing Rate, and Skin Temperature throughout Pregnancy and the Impact of Emotions—A Longitudinal Evaluation Using a Sensor Bracelet
Source: Sensors (Basel). 2023 Jul 23;23(14):6620. doi: 10.3390/s23146620 (PMC10385491; doi:10.3390/s23146620)

Wrist skin temperature °C

**Model 1: anxiety**	
Gestational age (weeks)	**-0.03 [-0.03, -0.02]**
Anxious (sometimes to always)	**-0.15 [-0.22, -0.07]**
**Model 2: stress**	
Gestational age (weeks)	**-0.02 [-0.03, -0.01]**
Stressed (most of the time to always)	0.01 [-0.08, 0.09]
**Model 3: tiredness**	
Gestational age (weeks)	**-0.02 [-0.03, -0.01]**
Tired (most of the time to always)	**0.11 [0.03, 0.19]**
**Model 4: sensitivity**	
Gestational age (weeks)	**-0.02 [-0.03, -0.01]**
Sensitive (most of the time to always)	0 [-0.13, 0.13]
**Model 5: unmotivated**	
Gestational age (weeks)	**-0.02 [-0.03, -0.01]**
Unmotivated (most of the time to always)	-0.06 [-0.15, 0.03]
**Model 6: calm**	
Gestational age (weeks)	**-0.02 [-0.03, -0.01]**
Calm (sometimes to never)	-0.02 [-0.12, 0.08]
**Model 7: energized**	
Gestational age (weeks)	**-0.02 [-0.04, -0.01]**
Energized (sometimes to never)	-0.05 [-0.23, 0.1]
**Model 8: happiness**	
Gestational age (weeks)	**-0.02 [-0.03, -0.02]**
Happy (sometimes to never)	0.06 [-0.03, 0.14]

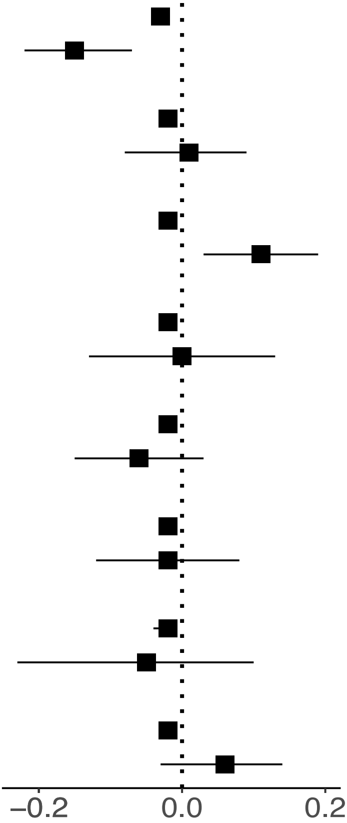

Supplement: Supplementary file 1 [file sensors-23-06620-s001.zip › Suppl. Figure S1d.pdf]
