# Supplementary material for: Changes in Heart Rate, Heart Rate Variability, Breathing Rate, and Skin Temperature throughout Pregnancy and the Impact of Emotions—A Longitudinal Evaluation Using a Sensor Bracelet
Source: Sensors (Basel). 2023 Jul 23;23(14):6620. doi: 10.3390/s23146620 (PMC10385491; doi:10.3390/s23146620)
Supplement: Supplementary file 1 [file sensors-23-06620-s001.zip › Suppl. Table S1.pdf]

Supp Table S1: Mean weekly measurements of the four basic physiological parameters during pregnancy.

Gestational Week	Wrist Skin Temperature		Breathing Rate		Heart Rate		Heart Rate Variability	
	Mean	SD	Mean	SD	Mean	SD	Mean	SD
8	35,40	0,60	14,55	0,12	61,47	1,41	67,95	5,08
9	36,01	0,43	13,44	1,49	64,83	2,61	60,97	2,98
10	36,54	0,33	12,34	0,15	65,63	2,06	60,54	4,69
11	36,50	0,27	15,27	3,34	64,71	2,48	55,19	5,32
12	36,33	0,26	15,74	3,17	67,42	3,11	48,17	4,80
13	36,08	0,45	16,21	2,99	62,60	5,30	62,01	18,96
14	36,02	0,48	16,25	2,82	62,23	6,06	63,32	19,12
15	35,96	0,44	16,15	3,11	62,89	6,21	58,65	14,68
16	35,94	0,54	16,06	2,87	64,04	7,85	55,80	13,05
17	35,98	0,43	16,18	2,46	65,66	9,24	53,77	11,67
18	35,95	0,49	15,77	2,46	66,13	9,69	50,86	11,84
19	35,94	0,43	15,96	2,24	65,86	9,83	51,41	11,43
20	35,89	0,43	15,88	2,48	65,48	9,05	52,52	10,78
21	35,85	0,50	15,80	1,96	64,34	8,56	53,59	10,23
22	35,68	0,61	16,13	2,14	63,95	10,24	50,85	10,35
23	35,76	0,52	15,67	2,07	65,16	10,18	52,06	11,35
24	35,71	0,49	16,05	2,14	65,68	10,16	52,01	12,57
25	35,79	0,44	15,42	1,77	66,57	8,29	51,29	19,15
26	35,66	0,53	15,22	1,74	65,74	9,39	49,66	8,82
27	35,73	0,52	15,17	1,95	65,80	10,06	50,30	9,61
28	35,67	0,55	15,25	2,11	65,74	9,80	48,98	9,43
29	35,62	0,49	15,29	2,26	66,75	10,15	51,42	11,30
30	35,69	0,47	14,93	2,38	64,06	9,70	50,44	11,61
31	35,60	0,48	15,08	2,23	64,73	10,38	50,57	9,20
32	35,53	0,46	14,98	1,93	64,39	9,86	50,83	10,00
33	35,59	0,42	15,30	2,26	66,03	9,54	51,00	11,44
34	35,47	0,60	15,53	2,31	66,83	12,06	50,89	11,35
35	35,35	0,67	14,99	2,22	67,12	10,57	51,45	10,33
36	35,30	0,88	14,48	2,14	63,23	13,70	54,11	10,23
37	35,23	0,92	14,87	2,16	63,81	10,67	56,64	11,41
38	35,42	0,58	14,85	2,05	62,81	10,04	59,36	10,51
39	35,67	0,84	13,56	0,99	64,13	4,06	57,55	13,95
40	35,57	0,54	14,48	0,15	63,49	0,66	57,31	3,90
